# Component- and dimension-level network associations between sleep quality and health-related quality of life in older adults with hypertension

**DOI:** 10.3389/fpubh.2026.1890897

**Published:** 2026-09-15

**Authors:** Xinyuan Sun, Yanan Guo, Baoyang Ding, Jun Hu

**Affiliations:** 1 School of Public Health, Shandong Second Medical University, Weifang, Shandong, China; 2 School of Health Management, Shandong University of Traditional Chinese Medicine, Jinan, Shandong, China

**Keywords:** depressive symptoms, health-related quality of life, hypertension, network analysis, older adults, sleep quality

## Abstract

**Background:**

Older adults with hypertension commonly experience poor sleep quality and reduced health-related quality of life (HRQoL). Previous studies have mainly examined these associations using total scores, providing limited insight into interactions between specific symptom dimensions. This study aimed to construct a component- and dimension-level network linking sleep quality and HRQoL and to compare network differences across depressive-symptom status.

**Methods:**

This study included 2,257 older adults. Sleep quality, HRQoL, and depressive symptoms were assessed using the B-PSQI, EQ-5D-5L, and PHQ-9, respectively. We estimated a component- and dimension-level network based on the EBICGlasso-regularized Gaussian graph model, calculated centrality and bridge centrality metrics, validated stability using the bootstrap method, and compared network differences between the depressive and non-depressive-symptom groups.

**Results:**

The final network included 10 nodes and 32 non-zero edges among 45 possible edges, with a density of 0.711. The strongest associations were between sleep efficiency and sleep duration (SE–ST, 0.793), sleep interruption and subjective sleep quality (SW–SQ, 0.583), and self-care and usual activities (SC–UA, 0.560). Usual activities (UA) showed the highest strength centrality and expected influence, followed by subjective sleep quality (SQ), self-care (SC), and sleep efficiency (SE). Sleep efficiency had the highest bridge expected influence, followed by subjective sleep quality. Centrality indices demonstrated good stability (CS = 0.75). No significant differences were found in network structure (M = 0.224, *P* > 0.05) or global strength (S = 0.119, *P* > 0.05); repeated 1:1 subsampling broadly supported these findings.

**Conclusion:**

Sleep components and HRQoL dimensions formed a closely connected network in older adults with hypertension. Usual activities showed the highest centrality, while sleep efficiency and subjective sleep quality showed prominent cross-community. These domains may be useful for screening or hypothesis generation but should not be interpreted as confirmed intervention targets.

## 1 Introduction

With the acceleration of population aging, the disease burden of hypertension among older adults continues to rise. China is undergoing rapid population aging. According to Seventh National Population Census shows that the population aged 60 and above has reached 264 million, and the proportion of the older adults (aged ≥60) in the global population is steadily increasing (1). Against this backdrop, hypertension has consequently become one of the most prevalent chronic diseases among older adults and currently affects about 245 million people in China (2, 3). Beyond increasing the risks of stroke, coronary heart disease, and renal dysfunction, hypertension can also impair daily functioning and overall health status in later life (4–6). Therefore, identifying patterns of health impairment in older hypertensive adults is an important issue in geriatric and public health research.

The adoption of a healthy lifestyle plays a pivotal role in preventing or delaying the onset of hypertension, thereby reducing cardiovascular risk(7, 8). Among them, sleep quality and health-related quality of life (HRQoL) serve as two critical entry points for understanding the disease burden in older patients with hypertension. Previous studies have shown that both insufficient and excessive sleep duration are associated with hypertension risk, adverse cardiovascular outcomes, and poorer functional status (9–11). Meanwhile, the decline in quality of life is also a central issue in the long-term management of hypertension. Hypertension may also impair mobility, self-care abilities, and daily activities, thereby reducing the independent living capacity and overall health perception of older patients (12). However, most existing studies treat sleep quality and HRQoL as aggregate variables, primarily employing total score comparisons, correlation analyses, or regression models to assess their linear relationships (13, 14). While this score-oriented approach captures overall correlations, it fails to elucidate specific connections between different sleep dimensions and HRQoL dimensions, nor does it facilitate identifying the most critical components requiring prioritized attention within the system.

Network analysis provides a more suitable framework for addressing this issue. Unlike traditional approaches that regard symptoms as indicators of an underlying disorder, network analysis conceptualizes symptoms or dimensions as interacting components within a system (14, 15). This method can identify central nodes and bridge nodes that connect different domains (14, 16), making it suitable for studying the relationship between sleep quality and HRQoL. Although recent studies have applied network analysis to psychological symptoms and life satisfaction in older hypertensive patients, research on the hierarchical relationship between sleep quality and HRQoL dimensions remains limited, especially studies that simultaneously incorporate specific sleep dimensions and HRQoL dimensions (17). Network methods focus on associations among components and dimensions rather than aggregate scores, enabling higher clinical precision in identifying associations among components and dimensions. In recent years, network analysis has been increasingly applied to mental disorders, chronic disease symptom systems, and health-related behavior research (14). However, evidence from network analyses regarding the hierarchical relationship between sleep quality and HRQoL in older hypertensive populations remains scarce. Ma's study has begun exploring relationships at the “symptom/dimension level,” but sleep has not been adequately integrated, nor has it been linked to HRQoL dimensions (17).

Depressive symptoms may modify the relationship between sleep quality and HRQoL. Depression is common among people with hypertension, particularly older adults, and is associated with sleep disturbance, functional impairment, and reduced quality of life (18–21). In the present study, depressive-symptom status was used as a stratification variable rather than included as a network node because the primary objective was to examine whether the conditional associations between sleep components and HRQoL dimensions differed between participants with and without clinically relevant depressive symptoms. This approach also allowed us to examine potential clinical heterogeneity within the hypertensive population (22, 23).

Previous studies have primarily examined sleep disorders, depression, and HRQoL using total scores or conventional regression models (10, 13, 14, 24). Consequently, the conditional associations between specific sleep components and HRQoL dimensions remain insufficiently understood. Although network analysis has been applied to psychological symptoms in older adults with hypertension, few studies have jointly examined sleep components and HRQoL dimensions or compared their network organization according to depressive-symptom status (17). A component- and dimension-level network may therefore help characterize prominent conditional associations and identify domains warranting further assessment and prospective investigation.

Therefore, this study aimed to estimate a component- and dimension-level network of sleep quality and HRQoL among older adults with self-reported physician-diagnosed hypertension in China. We examined node centrality and cross-community connectivity and compared the network structure between participants with and without clinically relevant depressive symptoms. These analyses were intended to identify potential screening or hypothesis-generating indicators for future longitudinal and interventional research.

## 2 Method

### 2.1 Participants and procedures

**Figure 1:**
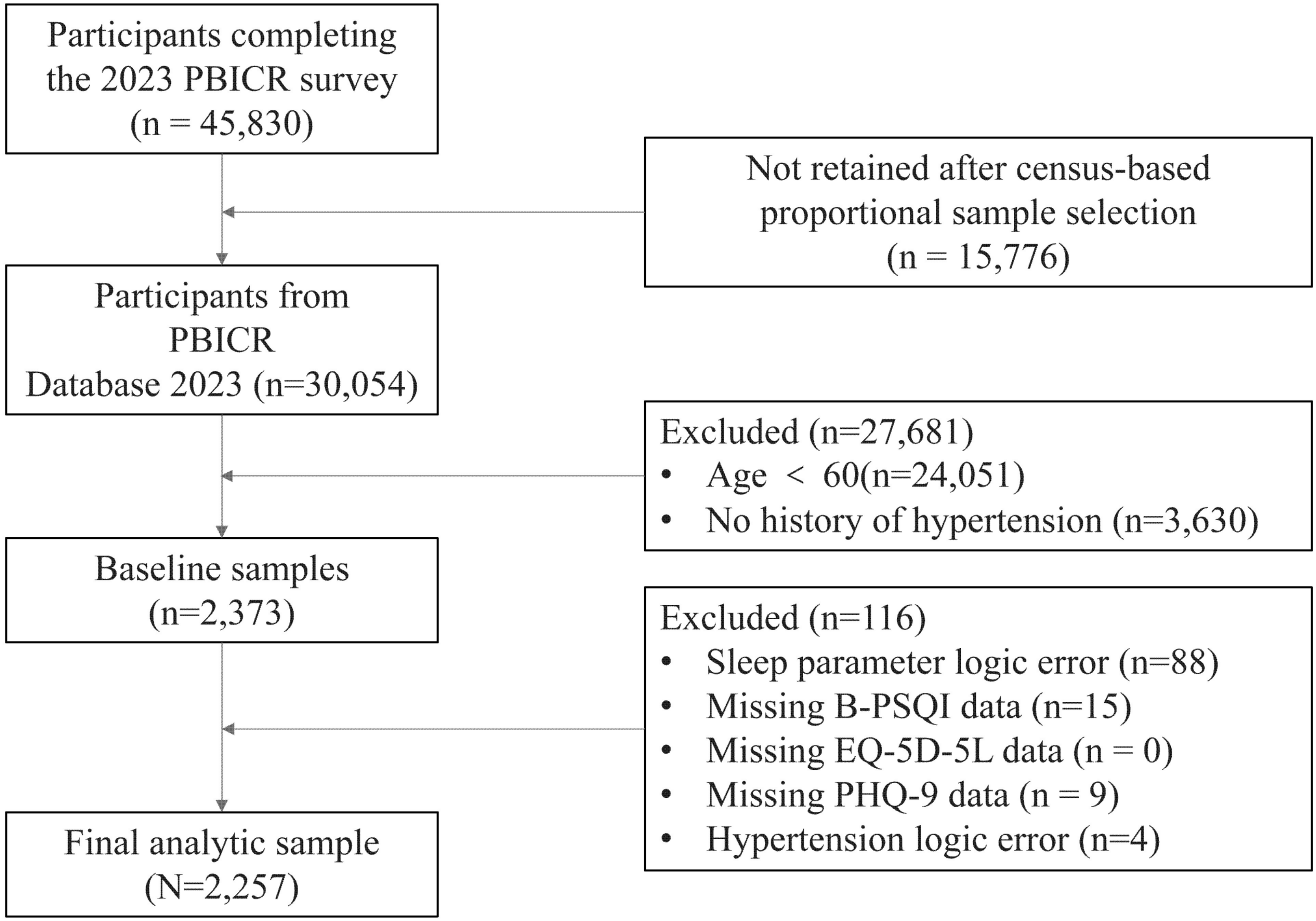
Participant selection process for this cross-sectional study of hypertensive individuals from a 2023 national cross-sectional survey in China.

The present study used data from the fourth wave of the Psychology and Behavior Investigation of Chinese Residents (PBICR), a comprehensive cross-sectional survey designed to reflect the demographic characteristics of the entire Chinese population. The survey was conducted from June 20 to August 31, 2023, following over 30 expert consultations and three rounds of preliminary research. It employed a design combining stratified sampling (ensuring equal probability across administrative regions) with quota sampling (allowing probability variations from the community/village level to the individual level), covering 150 cities across all provinces in China. Face-to-face interviews were conducted, yielding a total sample size of 45,830 participants. The sample was proportionally matched according to gender, age, and urban-rural distribution characteristics from the Seventh National Population Census, resulting in a final sample size of 30,054 participants. The survey underwent ethical review (SWYX: NO.2023-198). This study constitutes a targeted analysis of the large-scale survey, Participants were eligible if they were aged ≥60 years and reported having received a physician diagnosis of hypertension during the face-to-face survey. A total of 2,373 participants were screened. To ensure data quality, further analysis revealed inconsistencies in sleep data (*n* = 88), absence of B-PSQI data (*n* = 15), missing PHQ-9 data (*n* = 9), or inconsistent hypertension data (*n* = 4). The final analysis sample consisted of a total of 2,257 participants. Figure 1 shows the complete screening process.

### 2.2 Measurements

Demographic variables were collected from the participants, including gender, age, per capita monthly household income, living arrangement (single or married), marital status, education level, place of residence (urban/rural), and number of children.

### 2.3 Sleep quality assessment

Sleep quality was assessed using the Brief Pittsburgh Sleep Quality Index (B-PSQI) to evaluate respondents' sleep conditions over the past month. Following the methodology employed in previous PBICR homologous studies, the B-PSQI consists of five components: Sleep efficiency (SE), Sleep time (ST), Sleep awakenings (SW), Sleep latency (SL), and Subjective sleep quality (SQ). Each item is scored on a 0–3 point scale, with a total score range of 0–15 points; higher scores indicate poorer sleep quality. The Chinese version of the B-PSQI has demonstrated acceptable reliability and validity in the Chinese sample (25, 26).

#### 2.3.1 Quality of life assessment

This study employed the EuroQol Five Dimensions Questionnaire (EQ-5D-5L) to assess respondents ‘health-related quality of life (HRQoL). The questionnaire comprises five dimensions: Mobility (MA), Self-care (SC), Usual activities (UA), Pain/discomfort (PD), and Anxiety/depression (AD), each with five levels scored from 1 to 5, corresponding to no problems, slight problems, moderate problems, severe problems and extreme problems, respectively (27). The EQ-5D-5L has been widely used for life quality assessment, and its Chinese version has demonstrated acceptable structural validity and moderate-to-good test-retest reliability in the general Chinese population (28).

#### 2.3.2 Depressive symptom assessment

This study employed the Patient Health Questionnaire-9 (PHQ-9) to assess participants' depressive symptoms over the past 2 weeks. The scale comprises nine items: low mood, diminished interest, sleep problems, fatigue, changes in appetite, feelings of worthlessness, difficulty concentrating, psychomotor retardation or agitation, and suicidal ideation. Each item is scored on a four-point scale (0–3), with a total score ranging from 0 to 27, where higher scores indicate more severe depressive symptoms. The Cronbach's α coefficient for the full scale is 0.89. The original validation study demonstrated that a PHQ-9 score ≥10 exhibited an 88.0% sensitivity and specificity for screening major depressive disorder (29); the Chinese version of the PHQ-9 also showed good reliability and validity in the general Chinese population (30). Therefore, this study defined scores between 0–9 as indicating no significant depressive symptoms, while a score ≥10 indicated the presence of depressive symptoms.

### 2.4 Statistical analyses

Statistical analyses were performed in R version 4.5.3 using qgraph (version 1.9.8), bootnet (version 1.8), networktools 1.6.0, NetworkComparisonTest (version 2.2.3), and mgm (version 1.2.15) packages. Networks were estimated as regularized Gaussian graphical models using bootnet::estimateNetwork() with default = “EBICglasso”(31, 32). The five B-PSQI components and five EQ-5D-5L dimensions were treated as ordinal variables. Correlation matrices were computed using corMethod = “cor_auto”; all nodes were identified as ordinal and polychoric correlations were therefore used. (31). The EBIC hyperparameter was set to γ = 0.50 (31). Complete-case analysis was applied to hypertension status, depressive-symptom classification, and all network nodes, with no imputation.

Nodes represented B-PSQI components and EQ-5D-5L dimensions, and edges represented regularized partial correlations after controlling for all other nodes (32). Node centrality was summarized using strength and expected influence (EI) (33, 34). Bridge strength and one-step bridge expected influence were used to assess cross-community connectivity (16), with the B-PSQI components and EQ-5D-5L dimensions defined *a priori* as two communities. Node predictability was estimated using a pairwise categorical mixed graphical model in mgm with k = 2 and EBIC γ = 0.50 (35, 36). Because all nodes were categorical, predictability was expressed as normalized correct classification (nCC), and interpreted as in-sample predictability (35). Comparisons between four- and five-level nodes were made cautiously.

Two-sided Wilcoxon rank-sum tests compared node scores between participants with and without clinically relevant depressive symptoms. Because tied values were present, asymptotic *P* values were calculated using exact = FALSE. The resulting *P* values were adjusted across the 10 node-level tests using the Benjamini–Hochberg procedure.

Edge-weight accuracy was assessed using 1,000 nonparametric bootstrap samples, and centrality stability was evaluated using 1,000 case-dropping bootstrap samples (37). Strength, EI, bridge strength, and bridge expected influence were evaluated in the full network, whereas strength and EI were evaluated in each subgroup. CS coefficients below 0.25 were considered inadequate, whereas values above 0.50 were preferred (37).

Subgroup networks were compared using the Network Comparison Test with 1,000 permutations for independent weighted networks (38). Network structure, global strength, individual edges, strength, and EI were examined. Global strength was defined as the sum of absolute edge weights (38). Benjamini–Hochberg correction was applied to individual-edge and node-level centrality tests (38), and correlation matrices were made positive definite when required.

To assess construct overlap, the full and subgroup networks were re-estimated after excluding the EQ-5D-5L anxiety/depression node, while retaining the original PHQ-9 classification and analytical settings (32). To assess unequal subgroup sizes, 100 repeated 1:1 subsampling analyses were conducted. In each iteration, 409 participants were randomly selected without replacement from the non-depressive-symptom group and compared with all 409 participants in the depressive-symptom group. Each comparison used the primary network settings and 1,000 NCT permutations. NCT P values and Spearman correlations for strength, EI, and edge weights were summarized descriptively. To assess estimator sensitivity, the full network was re-estimated using EBIC tuning parameters of γ = 0.25, 0.50, and 0.75. Network density, edge weights, and strength and expected-influence rankings were compared across models. The significance level was set at α = 0.05.

## 3 Result

### 3.1 Study sample

This study enrolled 2,257 older hypertensive patients in China, with an average age of 71.11 years (standard deviation = 6.754). Among them, there were 1,187 males and 1,070 females. Other demographic characteristics, including per capita monthly household income, living status, marital status, education level, urban/rural residence, and number of children, are presented in Table 1. The mean total scores of the B-PSQI were 4.67 (SD = 3.16), those of the EQ-5D-5L were 7.51 (SD = 3.16), and those of the PHQ-9 were 5.44 (SD = 4.90). Additionally, 409 participants exhibited significant depressive symptoms (18.12%, PHQ-9 ≥10). The mean scores, standard deviations, and symptom predictability of sleep quality across HRQoL items are shown in Table 2. The predictability results indicated that daily activities (UA) showed the highest in-sample nCC (0.555), followed by mobility (MA, 0.471), self-care (SC, 0.415), and pain/discomfort (PD, 0.396). sleep latency (SL, 0.142) and sleep time (ST, 0.144) showed the lowest nCC values. Comparisons between nodes with different numbers of response categories should be interpreted cautiously.

**Table 1:** Demographic characteristics of the sample (*N* = 2,257).

Variable	Mean (SD) or *n* (%)
Gender	
Male	1,187 (52.6%)
Female	1,070 (47.4%)
Age, year	71.11 (6.75)
60–69years	995 (44.1%)
70–79years	1,015 (45.0%)
80–89years	230 (10.2%)
≥90	17 (0.75%)
Per capita monthly household income (RMB)	
≤ 2,000	535 (23.7%)
2,001–5,000	1,142 (50.6%)
5,001–9,000	424 (18.8%)
≥9,000	156 (6.9%)
Living alone status	
Living alone	384 (17.0%)
Not living alone	1,873 (83.0%)
Marital status	
Never married	59 (2.6%)
Married	1,745 (77.3%)
Divorced	53 (2.3%)
Widowed	400 (17.7%)
Education level	
No formal education	415 (18.4%)
Primary school	644 (28.5%)
Junior high school/technical school	654 (29.0%)
Senior high school and above	544 (24.1%)
Residence	
Urban	1,185 (52.5%)
Rural	1,072 (47.5%)
Number of children	
0	44 (1.9%)
1	559 (24.8%)
2	915 (40.5%)
≥3	679 (30.1%)
Missing	60 (2.7%)
B-PSQI score	4.67 (3.16)
EQ-5D-5L score	7.51 (3.16)
PHQ-9 score	5.44 (4.9)
0–9	1,848 (81.88%)
≥10	409 (18.12%)

**Table 2:** Descriptive statistics and network predictability of B-PSQI and EQ-5D-5L nodes.

Label	Items	Mean	SD	nCC
SL	Sleep latency	1.101	0.939	0.142
SW	Sleep wakefulness	1.304	1.043	0.305
SQ	Subjective sleep quality	1.186	0.733	0.232
SE	Sleep efficiency	0.623	1.006	0.245
ST	Sleep time	0.447	0.818	0.144
MA	Mobility	1.545	0.775	0.471
SC	Self-care	1.346	0.679	0.415
UA	Usual activities	1.422	0.688	0.555
PD	Pain/discomfort	1.787	0.790	0.396
AD	Anxiety/depression	1.415	0.671	0.202

B-PSQI, Brief Pittsburgh Sleep Quality Index; EQ-5D-5L, EuroQol 5-Dimension 5-Level; SD, Standard deviation.

### 3.2 Network structure

**Figure 2:**
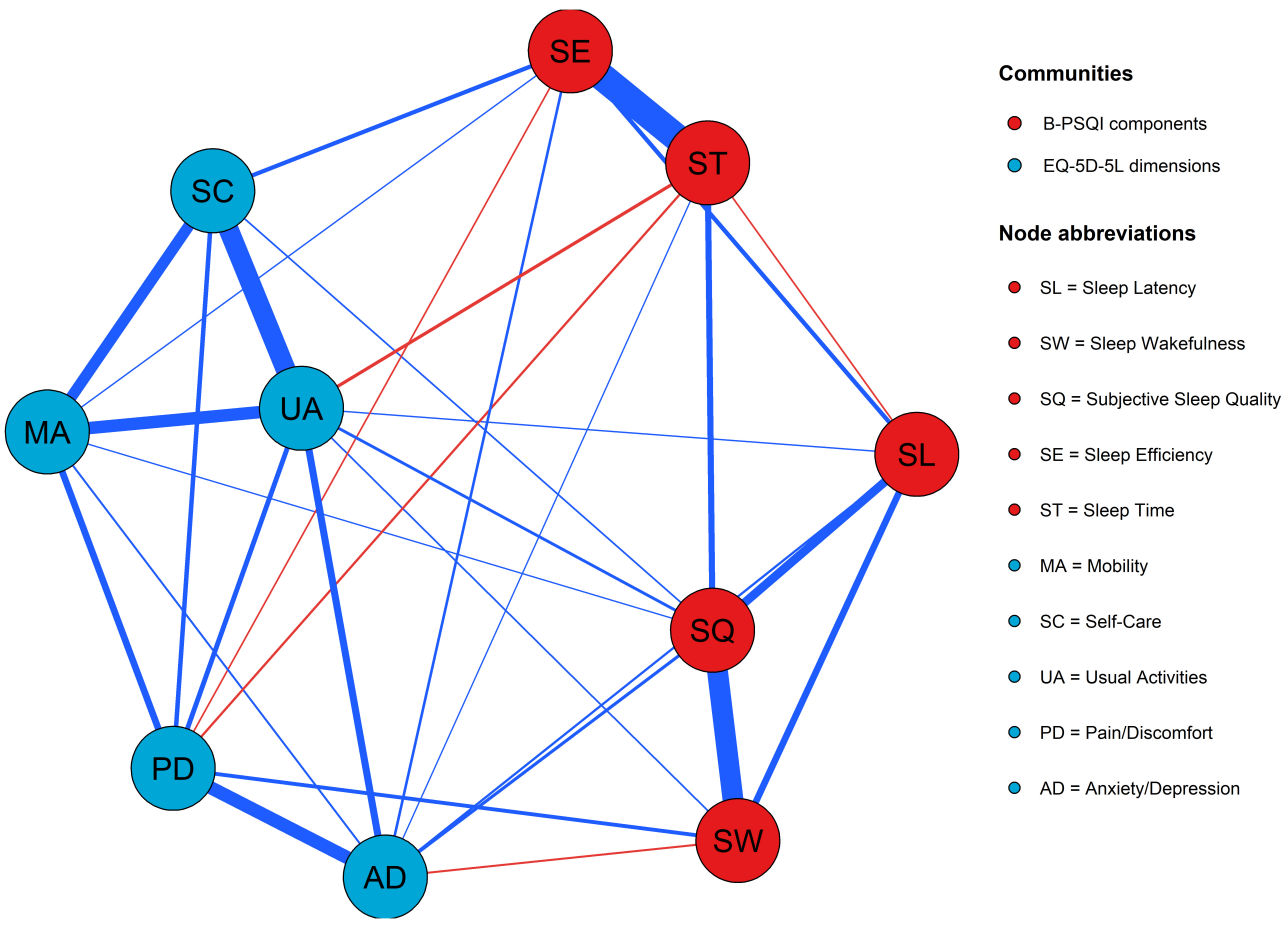
The network structure of sleep quality components and health-related quality of life dimensions among older adults with hypertension.

Figure 2 presents the 10-node component- and dimension-level network. Of the 45 possible edges, 32 were nonzero (density = 0.711), including 27 positive and 5 negative edges. The edge weight results indicate that the strongest edges are, in order: Sleep efficiency vs. Sleep time (SE–ST, weight = 0.793), Sleep wakefulness vs. Subjective sleep quality (SW–SQ, weight = 0.583), Self-care vs. Usual activities (SC–UA, weight = 0.560), Pain/discomfort vs. Anxiety/depression (PD–AD, weight = 0.361), and Mobility vs. Usual activities (MA–UA, weight = 0.336). These findings demonstrate strong local correlations both within the sleep dimension and within the HRQoL dimension.

**Figure 3:**
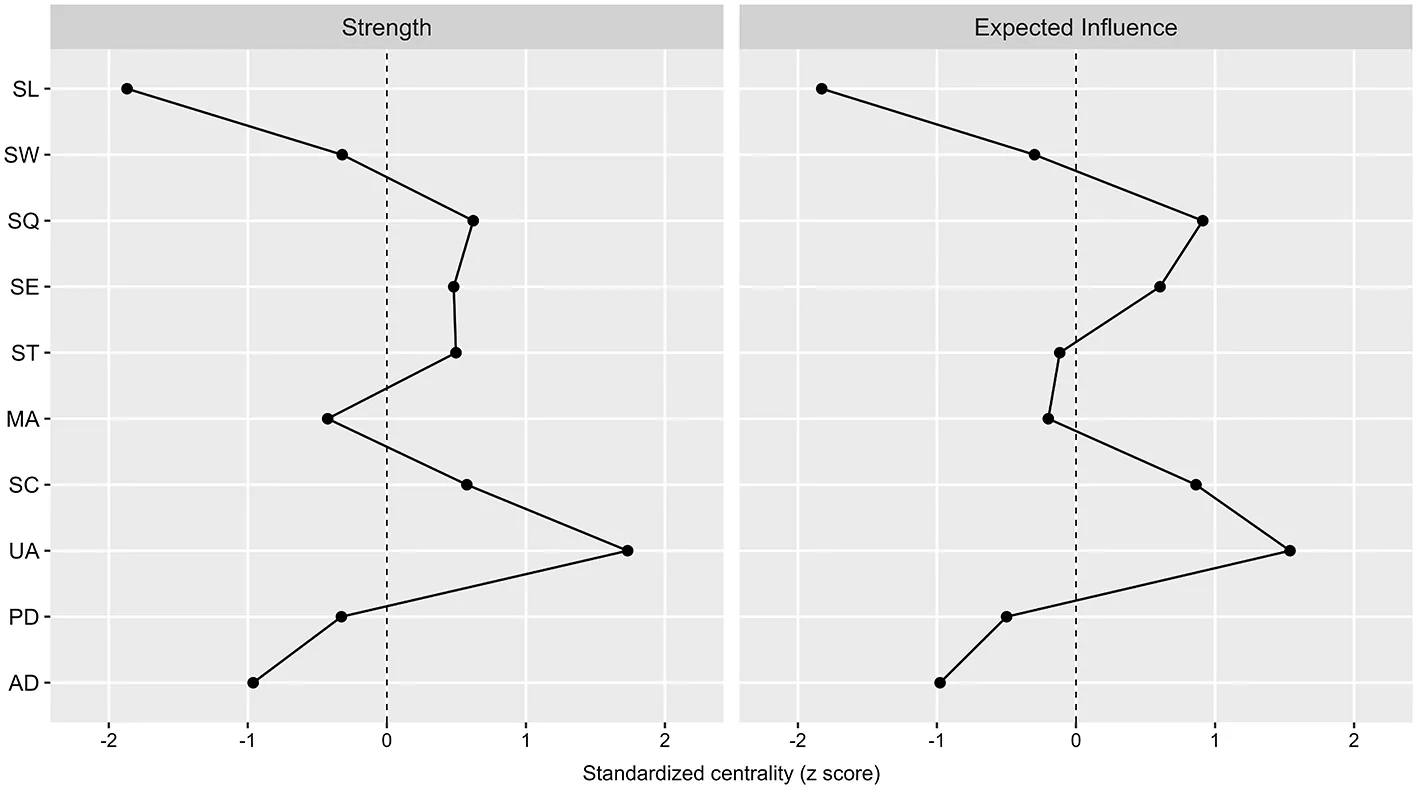
Strength and EI of the B-PSQI components and EQ-5D-5L dimensions. Values are presented as standardized z-scores.

**Figure 4:**
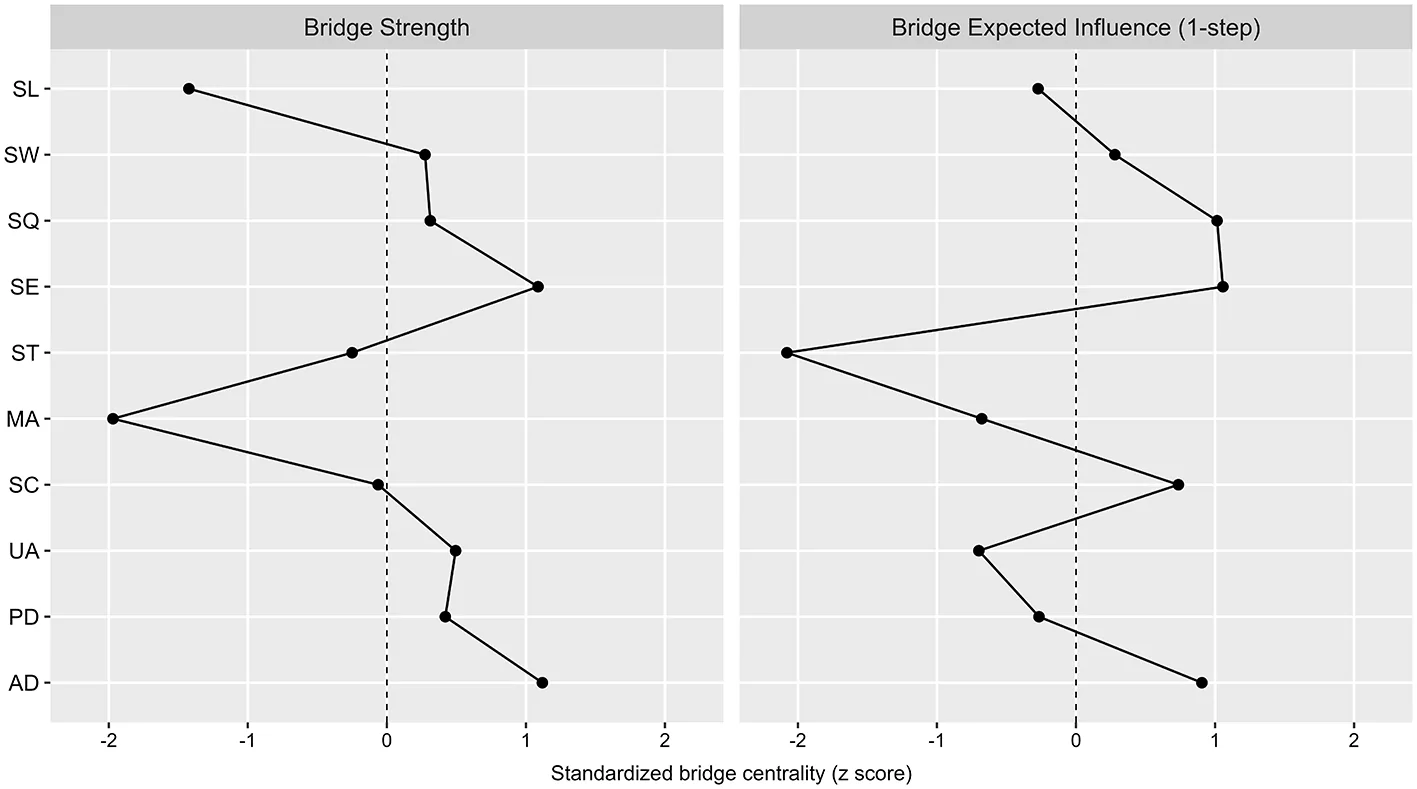
Bridge Strength and BEI of the B-PSQI components and EQ-5D-5L dimensions. Values are presented as standardized z-scores.

The centrality index of the network was assessed using strength and EI (Figure 3). The results demonstrated that UA (usual Activities) exhibited the highest strength (z = 1.731) and EI (z = 1.539) across the entire network; SQ (Subjective sleep quality) ranked second in both strength (z = 0.621) and EI (z = 0.912); SC (Self-care) and SE (Sleep efficiency) also showed elevated centrality metrics. In contrast, SL (Sleep latency) had the lowest values for strength (z = −1.869) and EI (z = −1.812), while AD (Anxiety/depression) also exhibited relatively low scores, indicating these nodes as the most peripheral symptoms within the network. This suggests that UA (Usual activities) is a highly influential functional node throughout the network, whereas SQ (Subjective sleep quality) ranked second for both indices (Figure 3). SL showed the lowest strength and expected influence. Bridge centrality analysis further revealed that SE (Sleep efficiency) had the highest BEI (0.117) (Figure 4), followed by SQ (Subjective sleep quality, 0.114) and AD (Anxiety/depression, 0.107), highlighting their relatively more critical bridging role between sleep quality dimensions and HRQoL dimensions. The prominent bridging function of SQ (Subjective sleep quality) underscores its status as a pivotal cross-dimensional node linking sleep quality and HRQoL dimensions.

### 3.3 Network stability

**Figure 5:**
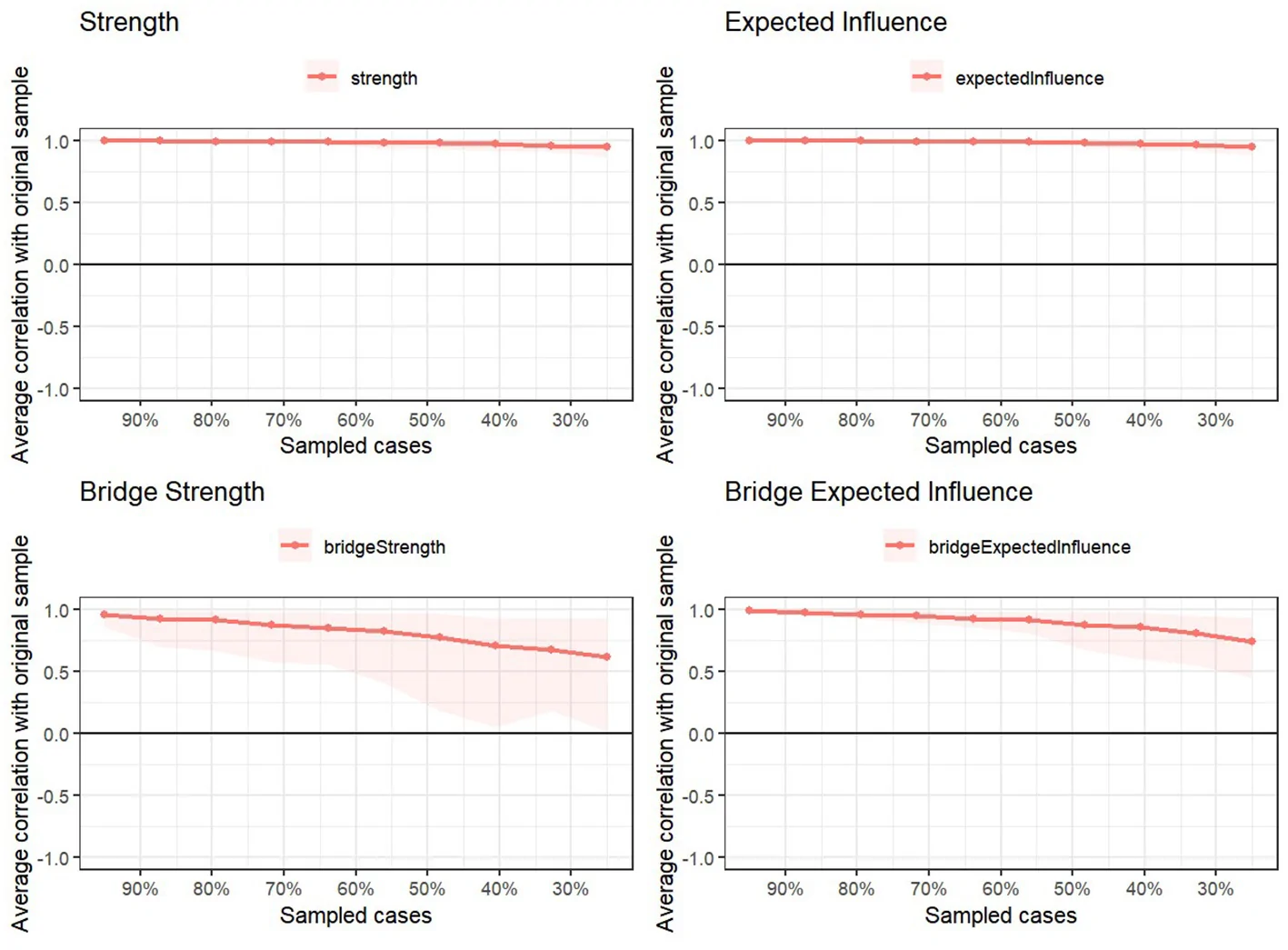
Case-dropping bootstrap stability of centrality and bridge-centrality indices. Lines represent the average correlations between centrality estimates from subsamples and those from the original sample.

As shown in Figure 5, the stability analysis results indicate that the centrality metrics primarily used for node explanation in this study demonstrate robust performance. Across the entire network, strength centrality, bridge strength centrality, EI centrality, and BEI centrality exhibited strong robustness. The CS coefficient of 0.75 indicated that the centrality estimates remained sufficiently correlated with those of the original sample after dropping substantial proportions of participants. Strength and expected influence showed high stability (CS = 0.75 for both), and one-step bridge expected influence showed acceptable stability (CS = 0.595). In contrast, bridge strength centrality showed insufficient stability (CS = 0.206) and was therefore not used for substantive interpretation. Consequently, this study primarily relies on BEI to interpret bridge nodes. Results regarding edge weight accuracy and difference testing are presented in Supplementary Figures. Supplementary Figure S1 displays the 95% confidence intervals for edge weights. Narrow confidence intervals for certain edges suggest precise weight estimates, although some overlap indicates difficulty in clearly distinguishing differences between edge weights. Difference estimates obtained through the bootstrap method are shown in Supplementary Figure S2. As indicated by the bootstrap difference tests (Supplementary Figure S3), edge weight differences were statistically significant in most comparisons.

### 3.4 Network differences by depressive status

**Figure 6:**
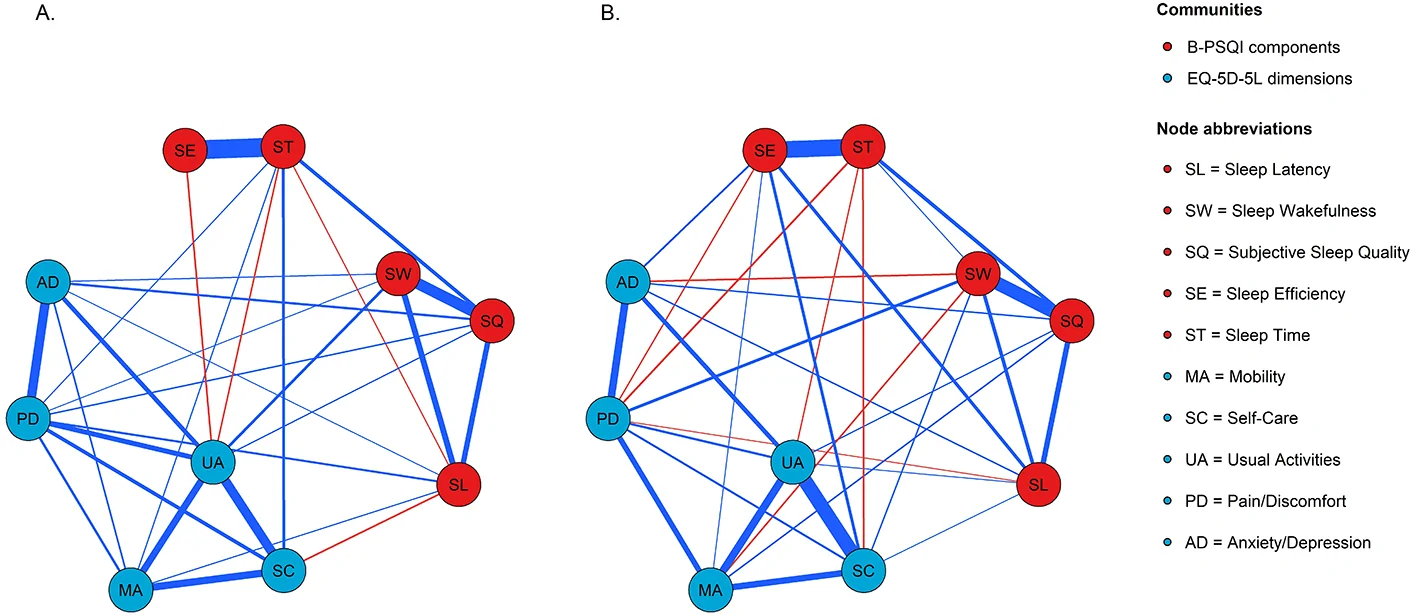
Quality of Sleep-HRQoL symptom network of hypertensive older adults divided by depression status. **(A)** With clinically relevant depressive symptoms. **(B)** Without clinically relevant depressive symptoms.

Figure 6 presents the networks for participants with and without clinically relevant depressive symptoms. The NCT showed no significant between-group differences in network structure (M = 0.224, *P* > 0.05) or global strength (S = 0.119, *P* > 0.05). The centrality invariance test demonstrated that the expected influence centrality of ST (Sleep time) was significantly higher in the non-depressive subgroup compared to the depressive subgroup, with a statistically significant difference (*P* < 0.05); differences in strength and EI for other nodes were not statistically significant. Additionally, the Wilcoxon rank-sum test with Benjamini–Hochberg correction revealed significant differences across all 10 nodes between the two groups, with the most notable differences observed for AD (Anxiety/depression), UA (Usual activities), SC (Self-care), MA (Mobility), and PD (Pain/discomfort). All 10 node-score distributions differed significantly between groups after Benjamini–Hochberg correction, with generally higher scores in participants with clinically relevant depressive symptoms (Supplementary Figure S4). Subgroup stability analysis further showed that the strength (CS = 0.516) and EI (CS = 0.594) metrics in the depressive-symptom group maintained acceptable stability, whereas the corresponding indicators in the non-depressive group exhibited higher stability (CS = 0.75). After excluding the EQ-5D-5L anxiety/depression dimension, the nine-node network preserved the strongest edges and centrality rankings (strength ρ = 0.933; EI ρ = 0.983), with no significant group differences in network structure (M = 0.227, *P* = 0.141), global strength (S = 0.029, *P* = 0.926), or individual edges after Benjamini–Hochberg correction (Supplementary Tables S2–S3 and Supplementary Dataset S1). In the repeated 1:1 subsampling analysis, the median P values were 0.158 (IQR, 0.050–0.361) for network structure and 0.421 (IQR, 0.084–0.733) for global strength; 25% and 20% of the 100 iterations yielded *P* < 0.05, respectively. Median rank correlations between the subsampled and full non-depressive networks were 0.867 for strength, 0.915 for expected influence, and 0.803 for edge weights, indicating broad but incomplete robustness to unequal group sizes.

### 3.5 Estimator sensitivity

Across γ = 0.25, 0.50, and 0.75, the networks contained 33, 32, and 28 non-zero edges, with densities of 0.733, 0.711, and 0.622, respectively. Compared with the primary model (γ = 0.50), edge-weight correlations remained high (Spearman ρ = 0.996 and 0.968), as did strength-ranking correlations (ρ = 1.000 and 0.952). Expected-influence rankings were unchanged, and UA, SQ, and SC consistently ranked highest. These findings indicate that the principal network results were robust to the EBIC tuning parameter (Supplementary Table S4).

## 4 Discussion

Based on a sample of older hypertensive patients in China, this study delineated the network structure between sleep quality and HRQoL at the component and dimension level and further compared network differences under varying depressive-symptom status. First, the study found that the entire network is predominantly characterized by positive connections with high density, suggesting that impaired sleep and declined HRQoL tended to co-occur rather than occur independently. The relatively high network density may reflect a combination of substantive associations among sleep and HRQoL domains, measurement overlap between closely related components within the same instrument, and the increased precision associated with the large sample size. Although EBICglasso regularization reduces weak and potentially spurious associations, a large sample may allow some small but stable conditional associations to be retained. Therefore, the observed density should not be interpreted as evidence of pervasive causal interdependence among all nodes. Second, UA (Usual activities) showed the highest centrality in the overall network, while SE (Sleep efficiency) showed the highest bridge expected influence between the sleep-quality and HRQoL communities (17, 39, 40).

In network analysis, the distribution of the strongest edges carries clear clinical implications. The strongest connection between SE (Sleep efficiency) and ST (Sleep time) suggests that sleep continuity and actual sleep acquisition at night may constitute critical components of sleep impairment in older individuals with hypertension. However, the strong SE–ST edge may partly reflect shared measurement content or computational dependence within the B-PSQI, because sleep efficiency is closely related to the amount of time spent asleep. Similarly, the strong SC–UA edge may partly reflect conceptual proximity within the EQ-5D-5L, as both dimensions assess limitations in daily functioning. These within-instrument edges may therefore represent both substantive conditional associations and measurement overlap and should not be interpreted as evidence of distinct causal pathways. This pattern is largely consistent with observations of different dimensions of sleep quality in previous hypertension studies and aligns with recent research and international position papers, which emphasize that sleep is not merely a marginal lifestyle factor in hypertension but rather a vital management component closely associated with nocturnal blood pressure regulation and cardiovascular risk (41–43). Another prospective cohort study based on the CLHLS further demonstrated that a healthy sleep pattern characterized by adequate sleep duration and good sleep quality is associated with a lower risk of hypertension in China's older adult population (44). Luo's research also demonstrates that the subjective perception of older adults is inextricably linked to their health status (45). Against this backdrop, the conditional associations among SE (Sleep efficiency), Sleep time (ST), and SQ (Subjective sleep quality) suggest that these related sleep components may warrant concurrent assessment rather than being interpreted in isolation (42, 44). However, their temporal and causal relationships cannot be determined from the present cross-sectional network.

One of the most significant findings of this study is the central role of UA (Usual activities), while SC (Self-care) also exhibits relatively high centrality. Unlike approaches that focus solely on emotional or somatic symptoms, these results indicate that when sleep quality and HRQoL are examined within the same network, functional capacity is more likely to occupy a central position throughout the system. This finding is particularly relevant for older hypertensive patients: hypertension-related blood pressure fluctuations, arterial stiffness, impaired autonomic regulation, and potential symptoms such as dizziness, fatigue, or orthostatic hypotension during antihypertensive treatment can directly impair daytime activity and daily functioning. These clinical characteristics may help explain why usual activities showed a central position in the estimated network, although the present analysis cannot determine whether functional limitations precede or result from sleep-related impairment (46, 47). Such as diabetes, COPD, or coronary disease, where fatigue, emotional issues, pain, or respiratory symptoms often occupy central positions in the network (48). The prominent centrality of UA (Usual activities) in this study suggests that the network structure in older hypertensive patients may more prominently reflect the core issue of impaired physical function. Previous studies have demonstrated that self-care behaviors, subjective health status, and social support are key determinants of health-related quality of life in older hypertensive patients, yet their self-care levels are often suboptimal (49). The higher centrality of SC (Self-care) further underscores the pivotal role of self-management capabilities in this population. Large-scale studies have shown a positive correlation between physical activity and HRQoL in older adults, with this relationship partially mediated by reduced depressive burden (50). Based on the findings of this study, UA (Usual activities) and SC (Self-care) should not be regarded merely as downstream outcomes; they may also represent critical dimensions worthy of attention when understanding the relationship between sleep impairment and decreased HRQoL in older hypertensive patients. However, whether these aspects constitute directional intervention targets requires further validation through longitudinal and intervention studies. From an assessment perspective, these findings support considering daily functional capacity and self-management limitations alongside blood pressure status in multidimensional geriatric evaluation (49, 50). The bridging role of SE (Sleep efficiency) also warrants emphasis, particularly its significant position within and across sleep dimensions. As a key indicator reflecting sleep continuity, sleep efficiency may more directly reflect the structural characteristics of sleep and its physiological recovery functions. Research by Oliveros et al. indicates that older hypertensive patients often exhibit abnormal nocturnal blood pressure regulation and autonomic nervous system dysfunction, factors that may be associated with reduced sleep efficiency and impaired sleep structure, thereby adversely affecting daytime functional status and quality of life (46). Concurrently, SQ (Subjective sleep quality) demonstrates strong connectivity within the network. Previous network studies involving older adults have frequently identified bridge nodes as tension, uncontrollable worry, or specific sleep disorder symptoms within the depression-anxiety-sleep connectivity network (17, 39, 40). However, in this study, sleep efficiency showed the highest cross-community connectivity, while subjective sleep quality also occupied a prominent bridge position. Subjective sleep quality may capture respondents' overall perception of multiple sleep-related experiences, including sleep latency, awakenings, efficiency, and duration, and may therefore serve as a useful patient-reported indicator of perceived sleep burden. Its relationship with daytime functioning should be considered hypothesis-generating and requires further longitudinal validation (42, 44, 51).

The full-sample NCT showed no significant differences in network structure or global strength between depressive-symptom groups. Repeated 1:1 subsampling reproduced these null findings in 75% and 80% of iterations, respectively, while centrality and edge-weight rankings remained generally stable. Thus, the subgroup findings were broadly, but not uniformly, robust and should be interpreted cautiously because of sampling variability.

The full and nine-node sensitivity analyses showed no significant differences in overall network structure or global strength between depressive-symptom groups. Excluding the anxiety/depression node preserved the strongest edges, the central role of usual activities, and the overall centrality rankings, suggesting that the principal structural findings were not primarily driven by construct overlap. However, the ordering of some bridge nodes and isolated node-level expected-influence results varied; these subgroup findings should therefore be interpreted as exploratory.

In this cross-sectional component- and dimension-level network, usual activities occupied the most central position, whereas sleep efficiency and subjective sleep quality showed relatively high cross-community connectivity (Figures 3, 4). Consistent with previous evidence that usual activities are an important HRQoL dimension in older adults, limitations in usual activities may be considered a potential indicator of broader functional burden. Similarly, sleep efficiency and subjective sleep quality should be assessed concurrently when sleep and HRQoL problems coexist (12, 42, 44, 52, 53). However, edges in a Gaussian graphical model and the resulting centrality and bridge-centrality indices describe conditional network positions and do not establish temporal precedence, causal influence, treatment responsiveness, or intervention efficacy (14, 16, 32, 54). Accordingly, these components and dimensions should be regarded as potential screening or hypothesis-generating indicators rather than confirmed intervention targets (14, 15, 32). Longitudinal and interventional studies are needed to determine whether changes in these domains precede or produce changes in other network components (14, 55, 56).

The strengths of this study include its large sample size and representative research cohort, which examined the relationship between sleep quality and HRQoL at the component and dimension level rather than solely at the total score level. This approach enabled us to identify nodes with prominent centrality or bridge centrality, particularly UA (Usual activities) and SQ (Subjective sleep quality). Furthermore, the study compared network structural differences according to depressive-symptom status, thereby supplementing evidence of clinical heterogeneity among older hypertensive patients. Additionally, the primary explanatory indicators used—including strength, bridge strength, EI, and BEI—all demonstrated acceptable to high stability, enhancing the credibility of the core findings. However, several limitations remain. First, the cross-sectional design precludes causal inference; network edges should be interpreted as conditionally correlated rather than directional pathways. Second, all variables were self-reported, potentially susceptible to recall bias and reporting bias. In addition, the PHQ-9-based grouping variable and the EQ-5D-5L anxiety/depression dimension have partially overlapping content. Although excluding the anxiety/depression node produced similar overall findings, measurement overlap cannot be completely excluded. Measurement overlap may also have contributed to strong within-instrument edges, particularly SE–ST within the B-PSQI and SC–UA within the EQ-5D-5L. Third, while key centrality metrics exhibited good stability, indicators such as Betweenness and Closeness showed limited consistency and thus were not prioritized for explanation. The smaller depressive-symptom group may also have reduced estimation precision; although repeated balanced subsampling broadly supported the primary findings, some sensitivity to sample composition remained. The study sample was drawn from older hypertensive individuals, so the findings may not be directly generalizable to other age groups or chronic disease populations. Finally, the network did not incorporate several important demographic and clinical factors, including comorbidity burden, antihypertensive medication use, and sleep apnea risk; therefore, residual confounding cannot be excluded. Future studies should incorporate these factors and objective sleep measures using longitudinal and multimodal designs.

## 5 Conclusion

In summary, sleep quality components and HRQoL dimensions formed a densely connected conditional-association network in older adults with self-reported physician-diagnosed hypertension. Usual activities showed the highest centrality, whereas sleep efficiency and subjective sleep quality showed relatively high bridge expected influence. Overall network structure and global strength did not differ significantly by depressive-symptom status, although the subgroup estimates showed some sensitivity to sample composition. Usual activities, sleep efficiency, and subjective sleep quality may serve as potential screening or hypothesis-generating indicators; however, the cross-sectional design does not establish them as causal or effective intervention targets. Longitudinal and interventional studies are required to determine their temporal and therapeutic relevance. The principal network findings remained broadly consistent after excluding the EQ-5D-5L anxiety/depression dimension.

## Data Availability

The data analyzed in this study is subject to the following licenses/restrictions: submission or other non-public occasions related to academic matters. Requests to access these datasets should be directed to Xinyuan Sun, sxy20010108@163.com.
