# Supplementary material for: Component- and dimension-level network associations between sleep quality and health-related quality of life in older adults with hypertension
Source: Front Public Health. 2026 Sep 15;14:1890897. doi: 10.3389/fpubh.2026.1890897 (PMC13588788; doi:10.3389/fpubh.2026.1890897)
Supplement: Supplementary file 1 [file Supplementary_file_1.docx]

Supplementary Figure S 1 Bootstrapped confidence intervals of edge weights

Supplementary Figure S 2 Estimation of edge weight difference by bootstrapped difference test

Supplementary Figure S 3 Nonparametric bootstrapped difference test

Supplementary Figure S 4 Node-score distributions by depressive-symptom status. Boxplots compare participants with (n = 409) and without (n = 1,848) clinically relevant depressive symptoms. All comparisons were significant after Benjamini–Hochberg correction.

Supplementary Table S 1 Regularized partial-correlation edge weights in the 10-node network

|  | **Node1** | **Node2** | **Edge_weight** | **Absolute_weight** | **Direction** | **Nonzero** |
| --- | --- | --- | --- | --- | --- | --- |
| **10** | SE | ST | 0.792881108 | 0.792881108 | Positive | TRUE |
| **3** | SW | SQ | 0.583116477 | 0.583116477 | Positive | TRUE |
| **28** | SC | UA | 0.559507365 | 0.559507365 | Positive | TRUE |
| **45** | PD | AD | 0.360544728 | 0.360544728 | Positive | TRUE |
| **27** | MA | UA | 0.336137711 | 0.336137711 | Positive | TRUE |
| **21** | MA | SC | 0.298116286 | 0.298116286 | Positive | TRUE |
| **2** | SL | SQ | 0.22173313 | 0.22173313 | Positive | TRUE |
| **44** | UA | AD | 0.177426486 | 0.177426486 | Positive | TRUE |
| **34** | MA | PD | 0.164486259 | 0.164486259 | Positive | TRUE |
| **1** | SL | SW | 0.156498749 | 0.156498749 | Positive | TRUE |
| **9** | SQ | ST | 0.141581971 | 0.141581971 | Positive | TRUE |
| **36** | UA | PD | 0.110693598 | 0.110693598 | Positive | TRUE |
| **35** | SC | PD | 0.095930225 | 0.095930225 | Positive | TRUE |
| **19** | SE | SC | 0.088305741 | 0.088305741 | Positive | TRUE |
| **4** | SL | SE | 0.086841642 | 0.086841642 | Positive | TRUE |
| **30** | SW | PD | 0.076878447 | 0.076878447 | Positive | TRUE |
| **26** | ST | UA | -0.057400706 | 0.057400706 | Negative | TRUE |
| **39** | SQ | AD | 0.053785913 | 0.053785913 | Positive | TRUE |
| **24** | SQ | UA | 0.047368539 | 0.047368539 | Positive | TRUE |
| **40** | SE | AD | 0.040381539 | 0.040381539 | Positive | TRUE |
| **37** | SL | AD | 0.030027007 | 0.030027007 | Positive | TRUE |
| **33** | ST | PD | -0.025733883 | 0.025733883 | Negative | TRUE |
| **38** | SW | AD | -0.021951023 | 0.021951023 | Negative | TRUE |
| **42** | MA | AD | 0.021102069 | 0.021102069 | Positive | TRUE |
| **32** | SE | PD | -0.016526594 | 0.016526594 | Negative | TRUE |
| **23** | SW | UA | 0.013599043 | 0.013599043 | Positive | TRUE |
| **7** | SL | ST | -0.010394213 | 0.010394213 | Negative | TRUE |
| **18** | SQ | SC | 0.008582515 | 0.008582515 | Positive | TRUE |
| **41** | ST | AD | 0.005136076 | 0.005136076 | Positive | TRUE |
| **14** | SE | MA | 0.004623838 | 0.004623838 | Positive | TRUE |
| **13** | SQ | MA | 0.004430353 | 0.004430353 | Positive | TRUE |
| **22** | SL | UA | 0.004180087 | 0.004180087 | Positive | TRUE |
| **5** | SW | SE | 0 | 0 | Zero | FALSE |
| **6** | SQ | SE | 0 | 0 | Zero | FALSE |
| **8** | SW | ST | 0 | 0 | Zero | FALSE |
| **11** | SL | MA | 0 | 0 | Zero | FALSE |
| **12** | SW | MA | 0 | 0 | Zero | FALSE |
| **15** | ST | MA | 0 | 0 | Zero | FALSE |
| **16** | SL | SC | 0 | 0 | Zero | FALSE |
| **17** | SW | SC | 0 | 0 | Zero | FALSE |
| **20** | ST | SC | 0 | 0 | Zero | FALSE |
| **25** | SE | UA | 0 | 0 | Zero | FALSE |
| **29** | SL | PD | 0 | 0 | Zero | FALSE |
| **31** | SQ | PD | 0 | 0 | Zero | FALSE |
| **43** | SC | AD | 0 | 0 | Zero | FALSE |

Supplementary Table S 2 NCT summary

| Outcome | N_valid | Median | Q1 | Q3 | Minimum | Maximum | Proportion_P_below_0_05 |
| --- | --- | --- | --- | --- | --- | --- | --- |
| Network-structure P value | 100 | 0.158 | 0.050 | 0.361 | 0.003 | 0.919 | 0.25 |
| Global-strength P value | 100 | 0.421 | 0.084 | 0.733 | 0.001 | 1 | 0.2 |
| Network M | 100 | 0.247 | 0.207 | 0.293 | 0.132 | 0.369 |  |
| Global-strength S | 100 | 0.347 | 0.147 | 0.943 | 0.001 | 2.247 |  |
| Strength-rank rho: subsample versus full non-depressive group | 100 | 0.867 | 0.758 | 0.939 | 0.285 | 0.976 |  |
| EI-rank rho: subsample versus full non-depressive group | 100 | 0.915 | 0.879 | 0.952 | 0.709 | 1 |  |
| Edge-rank rho: subsample versus full non-depressive group | 100 | 0.803 | 0.765 | 0.845 | 0.521 | 0.915 |  |

Supplementary Table S 3 Node ranking frequency

| Node | Count_strength | Proportion_strength | Count_EI | Proportion_EI |
| --- | --- | --- | --- | --- |
| SL | 0 | 0 | 0 | 0 |
| SW | 12 | 0.12 | 2 | 0.02 |
| SQ | 40 | 0.4 | 77 | 0.77 |
| SE | 37 | 0.37 | 37 | 0.37 |
| ST | 40 | 0.4 | 2 | 0.02 |
| MA | 8 | 0.08 | 4 | 0.04 |
| SC | 75 | 0.75 | 80 | 0.8 |
| UA | 84 | 0.84 | 98 | 0.98 |
| PD | 4 | 0.04 | 0 | 0 |
| AD | 0 | 0 | 0 | 0 |
|  |  |  |  |  |

Supplementary Table S 4 Supplementary Table S4. Sensitivity of network estimates to the EBIC tuning parameter.

| EBIC γ | Non-zero edges | Density | Edge-weight ρ | Strength-rank ρ | EI-rank ρ |
| --- | --- | --- | --- | --- | --- |
| 0.25 | 33 | 0.733 | 0.996 | 1 | 1 |
| 0.5 | 32 | 0.711 | 1 | 1 | 1 |
| 0.75 | 28 | 0.622 | 0.968 | 0.952 | 1 |

Supplementary Dataset S1 Supplementary Dataset Iteration-level results of the repeated 1:1 random-subsampling sensitivity analysis.

| Repetition | Sampling_seed | NCT_seed | Network_M | Network_p | GlobalStrength_S | GlobalStrength_p | GlobalStrength_Depressive | GlobalStrength_NonDep_Subsample | Strength_rank_rho_Sub_vs_Full | EI_rank_rho_Sub_vs_Full | Edge_rank_rho_Sub_vs_Full | Top3_Strength_NonDep_Subsample | Top3_EI_NonDep_Subsample | Error |
| --- | --- | --- | --- | --- | --- | --- | --- | --- | --- | --- | --- | --- | --- | --- |
| 1 | 1235 | 101235 | 0.259485656564777 | 0.133866133866134 | 1.60892862346055 | 0.015984015984016 | 4.62487058933275 | 6.23379921279331 | 0.515151515151515 | 0.781818181818182 | 0.814136747634244 | SC;SQ;SW | SC;SQ;SE | NA |
| 2 | 1236 | 101236 | 0.269054453600896 | 0.0989010989010989 | 0.800341951219129 | 0.0589410589410589 | 4.62487058933275 | 5.42521254055188 | 0.939393939393939 | 0.963636363636364 | 0.728748347488965 | UA;SC;ST | UA;SQ;SC | NA |
| 3 | 1237 | 101237 | 0.267088383142754 | 0.151848151848152 | 0.939325015994657 | 0.227772227772228 | 4.62487058933275 | 5.56419560532741 | 0.793939393939394 | 0.878787878787879 | 0.846468017778045 | ST;UA;SQ | SQ;MA;UA | NA |
| 4 | 1238 | 101238 | 0.232784265490455 | 0.197802197802198 | 1.52484927120946 | 0.00899100899100899 | 4.62487058933275 | 6.14971986054222 | 0.6 | 0.951515151515151 | 0.914514822854099 | SC;UA;PD | UA;SC;SQ | NA |
| 5 | 1239 | 101239 | 0.21568244138717 | 0.335664335664336 | 0.776581622094217 | 0.121878121878122 | 4.62487058933275 | 5.40145221142697 | 0.903030303030303 | 0.927272727272727 | 0.77142280026985 | UA;SC;ST | SC;UA;SQ | NA |
| 6 | 1240 | 101240 | 0.23719551537587 | 0.174825174825175 | 0.108754194565363 | 0.756243756243756 | 4.62487058933275 | 4.73362478389812 | 0.963636363636364 | 0.951515151515151 | 0.792625077863871 | SC;UA;SE | UA;SC;SE | NA |
| 7 | 1241 | 101241 | 0.244109089680897 | 0.196803196803197 | 0.0442014070996386 | 0.922077922077922 | 4.62487058933275 | 4.58066918223311 | 0.806060606060606 | 0.975757575757576 | 0.753282615930043 | UA;SC;ST | UA;SC;SQ | NA |
| 8 | 1242 | 101242 | 0.18459537234112 | 0.53946053946054 | 0.160728389113421 | 0.759240759240759 | 4.62487058933275 | 4.78559897844617 | 0.878787878787879 | 0.975757575757576 | 0.855727690752326 | UA;ST;SC | UA;SQ;SE | NA |
| 9 | 1243 | 101243 | 0.223319696573377 | 0.302697302697303 | 0.0409392872988477 | 0.916083916083916 | 4.62487058933275 | 4.5839313020339 | 0.83030303030303 | 0.939393939393939 | 0.817414869007028 | SC;SQ;UA | SQ;UA;MA | NA |
| 10 | 1244 | 101244 | 0.196708247393642 | 0.531468531468531 | 0.186567581680161 | 0.707292707292707 | 4.62487058933275 | 4.81143817101291 | 0.757575757575757 | 0.939393939393939 | 0.779121466535648 | SC;ST;SE | SC;UA;SQ | NA |
| 11 | 1245 | 101245 | 0.303082472060729 | 0.051948051948052 | 0.46025308419435 | 0.450549450549451 | 4.62487058933275 | 5.0851236735271 | 0.757575757575757 | 0.83030303030303 | 0.818584664965758 | UA;ST;SE | UA;SE;SC | NA |
| 12 | 1246 | 101246 | 0.256982472114125 | 0.127872127872128 | 0.524812474145421 | 0.277722277722278 | 4.62487058933275 | 5.14968306347817 | 0.624242424242424 | 0.963636363636364 | 0.789033405163515 | UA;SQ;ST | UA;SQ;SE | NA |
| 13 | 1247 | 101247 | 0.214921463133048 | 0.352647352647353 | 0.185484708805015 | 0.678321678321678 | 4.62487058933275 | 4.43938588052774 | 0.951515151515151 | 0.890909090909091 | 0.823305792549498 | UA;SC;SQ | UA;SC;SQ | NA |
| 14 | 1248 | 101248 | 0.195863807289677 | 0.464535464535465 | 0.228747756612671 | 0.509490509490509 | 4.62487058933275 | 4.85361834594542 | 0.866666666666667 | 0.987878787878788 | 0.755777183336983 | UA;SC;SQ | UA;SC;SQ | NA |
| 15 | 1249 | 101249 | 0.253398274382676 | 0.10989010989011 | 0.567210205410628 | 0.221778221778222 | 4.62487058933275 | 5.19208079474338 | 0.927272727272727 | 0.866666666666667 | 0.895858078515382 | UA;SE;SC | UA;SC;SQ | NA |
| 16 | 1250 | 101250 | 0.261545619720568 | 0.0999000999000999 | 0.988016777453487 | 0.051948051948052 | 4.62487058933275 | 5.61288736678624 | 0.284848484848485 | 0.975757575757576 | 0.68734675907674 | PD;MA;ST | UA;SQ;SC | NA |
| 17 | 1251 | 101251 | 0.245763446495547 | 0.180819180819181 | 0.0612798599252748 | 0.86013986013986 | 4.62487058933275 | 4.68615044925803 | 0.903030303030303 | 0.854545454545454 | 0.796434922481564 | UA;SE;SC | UA;SC;SE | NA |
| 18 | 1252 | 101252 | 0.262506447668248 | 0.0919080919080919 | 0.47043051976023 | 0.307692307692308 | 4.62487058933275 | 5.09530110909298 | 0.781818181818182 | 0.890909090909091 | 0.789674895736819 | UA;SE;MA | UA;SE;SQ | NA |
| 19 | 1253 | 101253 | 0.20131149647324 | 0.413586413586414 | 0.21144164135212 | 0.589410589410589 | 4.62487058933275 | 4.41342894798063 | 0.963636363636364 | 0.951515151515151 | 0.868719586959769 | UA;SC;SQ | UA;SC;SQ | NA |
| 20 | 1254 | 101254 | 0.258956593197171 | 0.127872127872128 | 1.0212947460716 | 0.138861138861139 | 4.62487058933275 | 5.64616533540435 | 0.927272727272727 | 0.915151515151515 | 0.788695778545987 | UA;SC;ST | UA;SE;SQ | NA |
| 21 | 1255 | 101255 | 0.266775093081306 | 0.0739260739260739 | 0.529048681796143 | 0.21978021978022 | 4.62487058933275 | 5.15391927112889 | 0.636363636363636 | 0.975757575757576 | 0.68725661340137 | SC;ST;SE | UA;SC;SQ | NA |
| 22 | 1256 | 101256 | 0.321342402245286 | 0.023976023976024 | 0.168194809652437 | 0.626373626373626 | 4.62487058933275 | 4.45667577968031 | 0.915151515151515 | 0.963636363636364 | 0.753900448355679 | UA;SQ;SE | UA;SQ;SE | NA |
| 23 | 1257 | 101257 | 0.309788717809203 | 0.022977022977023 | 0.100299014323671 | 0.785214785214785 | 4.62487058933275 | 4.72516960365642 | 0.975757575757576 | 0.939393939393939 | 0.750577733427048 | UA;SC;SQ | SC;SQ;UA | NA |
| 24 | 1258 | 101258 | 0.226413404663302 | 0.235764235764236 | 0.403065974844592 | 0.277722277722278 | 4.62487058933275 | 5.02793656417734 | 0.963636363636364 | 0.963636363636364 | 0.815999731435477 | SC;UA;SE | SC;UA;SQ | NA |
| 25 | 1259 | 101259 | 0.305558017110796 | 0.037962037962038 | 1.81135745686892 | 0.011988011988012 | 4.62487058933275 | 6.43622804620167 | 0.733333333333333 | 0.939393939393939 | 0.868304520864962 | SC;SW;UA | UA;SC;SQ | NA |
| 26 | 1260 | 101260 | 0.282420210898147 | 0.050949050949051 | 1.21971151407329 | 0.027972027972028 | 4.62487058933275 | 5.84458210340605 | 0.951515151515151 | 0.903030303030303 | 0.746166491481163 | UA;SC;SE | SC;SQ;UA | NA |
| 27 | 1261 | 101261 | 0.299767385487664 | 0.038961038961039 | 0.675181694208813 | 0.110889110889111 | 4.62487058933275 | 5.30005228354157 | 0.975757575757576 | 0.903030303030303 | 0.773953974825486 | UA;SC;SE | UA;SE;SC | NA |
| 28 | 1262 | 101262 | 0.228137729886991 | 0.235764235764236 | 0.210219484409616 | 0.542457542457542 | 4.62487058933275 | 4.41465110492314 | 0.915151515151515 | 0.927272727272727 | 0.809210638137102 | UA;SC;SQ | UA;SC;SQ | NA |
| 29 | 1263 | 101263 | 0.277943714973159 | 0.106893106893107 | 1.44666359832223 | 0.0979020979020979 | 4.62487058933275 | 6.07153418765499 | 0.587878787878788 | 0.939393939393939 | 0.856538269555432 | SW;SE;UA | UA;SQ;SE | NA |
| 30 | 1264 | 101264 | 0.217032061968979 | 0.292707292707293 | 0.684122018451421 | 0.0839160839160839 | 4.62487058933275 | 5.30899260778417 | 0.503030303030303 | 0.939393939393939 | 0.844467305504392 | UA;MA;SW | UA;SQ;SE | NA |
| 31 | 1265 | 101265 | 0.305420932972256 | 0.028971028971029 | 0.605658925321189 | 0.247752247752248 | 4.62487058933275 | 5.23052951465394 | 0.806060606060606 | 0.842424242424242 | 0.787632603733047 | UA;SE;ST | UA;SE;SC | NA |
| 32 | 1266 | 101266 | 0.207113743666738 | 0.333666333666334 | 0.440303522956894 | 0.261738261738262 | 4.62487058933275 | 5.06517411228965 | 0.6 | 0.975757575757576 | 0.760267617350109 | MA;SC;ST | UA;SC;SQ | NA |
| 33 | 1267 | 101267 | 0.312957313690915 | 0.035964035964036 | 1.85099941788773 | 0.018981018981019 | 4.62487058933275 | 6.47587000722048 | 0.842424242424242 | 0.975757575757576 | 0.581509434216998 | ST;UA;SC | UA;SQ;SE | NA |
| 34 | 1268 | 101268 | 0.196332505964653 | 0.401598401598402 | 0.410738100156359 | 0.323676323676324 | 4.62487058933275 | 4.21413248917639 | 0.866666666666667 | 0.939393939393939 | 0.757597004545103 | UA;SQ;SC | UA;SQ;SC | NA |
| 35 | 1269 | 101269 | 0.274109928239501 | 0.0869130869130869 | 0.10970030576823 | 0.782217782217782 | 4.62487058933275 | 4.73457089510098 | 0.866666666666667 | 0.806060606060606 | 0.726301897120985 | SE;SC;UA | SC;UA;SE | NA |
| 36 | 1270 | 101270 | 0.292398947241636 | 0.0609390609390609 | 0.0977692801888868 | 0.862137862137862 | 4.62487058933275 | 4.72263986952164 | 0.939393939393939 | 0.915151515151515 | 0.852966536110044 | SC;UA;ST | SC;UA;SQ | NA |
| 37 | 1271 | 101271 | 0.300466857288147 | 0.031968031968032 | 0.964465085316579 | 0.045954045954046 | 4.62487058933275 | 5.58933567464933 | 0.684848484848485 | 0.878787878787879 | 0.810221780420364 | SW;SQ;SC | SQ;SC;UA | NA |
| 38 | 1272 | 101272 | 0.286767472303273 | 0.036963036963037 | 1.50440198329817 | 0.002997002997003 | 4.62487058933275 | 6.12927257263093 | 0.951515151515151 | 0.866666666666667 | 0.776743195041236 | SC;UA;SE | SC;UA;SQ | NA |
| 39 | 1273 | 101273 | 0.277604172538202 | 0.0729270729270729 | 0.301860316853209 | 0.496503496503497 | 4.62487058933275 | 4.92673090618596 | 0.854545454545454 | 0.939393939393939 | 0.788608898501812 | SC;SQ;ST | SC;SQ;UA | NA |
| 40 | 1274 | 101274 | 0.177515703364644 | 0.619380619380619 | 0.163476646999698 | 0.586413586413586 | 4.62487058933275 | 4.46139394233305 | 0.927272727272727 | 0.866666666666667 | 0.791565604794977 | UA;SC;ST | UA;SE;MA | NA |
| 41 | 1275 | 101275 | 0.159428888935725 | 0.739260739260739 | 0.0392738900315628 | 0.936063936063936 | 4.62487058933275 | 4.58559669930119 | 0.660606060606061 | 0.903030303030303 | 0.835782669001326 | UA;MA;SW | UA;MA;SQ | NA |
| 42 | 1276 | 101276 | 0.332950292928032 | 0.018981018981019 | 0.392449277591036 | 0.370629370629371 | 4.62487058933275 | 5.01731986692379 | 0.951515151515151 | 0.915151515151515 | 0.82654088894857 | SC;UA;SQ | SC;SQ;UA | NA |
| 43 | 1277 | 101277 | 0.235226381921043 | 0.241758241758242 | 0.952930784481411 | 0.148851148851149 | 4.62487058933275 | 5.57780137381416 | 0.915151515151515 | 0.878787878787879 | 0.664562518610846 | SE;SC;SQ | SC;UA;SE | NA |
| 44 | 1278 | 101278 | 0.318146160931173 | 0.024975024975025 | 0.282955696451282 | 0.384615384615385 | 4.62487058933275 | 4.90782628578403 | 0.903030303030303 | 0.975757575757576 | 0.735789687579311 | SC;UA;ST | UA;SQ;SE | NA |
| 45 | 1279 | 101279 | 0.207113743666738 | 0.363636363636364 | 0.286907828235068 | 0.541458541458541 | 4.62487058933275 | 4.91177841756782 | 0.939393939393939 | 0.951515151515151 | 0.832860991886849 | SC;UA;ST | SC;SQ;UA | NA |
| 46 | 1280 | 101280 | 0.268393839461669 | 0.0709290709290709 | 1.37330218150042 | 0.020979020979021 | 4.62487058933275 | 5.99817277083317 | 0.890909090909091 | 0.878787878787879 | 0.772575044471126 | UA;SE;ST | SC;SQ;SE | NA |
| 47 | 1281 | 101281 | 0.169446489009669 | 0.684315684315684 | 0.176719368475912 | 0.71028971028971 | 4.62487058933275 | 4.44815122085684 | 0.963636363636364 | 0.866666666666667 | 0.843161632307256 | UA;SC;SQ | UA;SC;SQ | NA |
| 48 | 1282 | 101282 | 0.23098439043467 | 0.250749250749251 | 0.0422688450599393 | 0.921078921078921 | 4.62487058933275 | 4.58260174427281 | 0.939393939393939 | 0.903030303030303 | 0.820256260846088 | UA;SE;SC | UA;SC;SE | NA |
| 49 | 1283 | 101283 | 0.207113743666738 | 0.356643356643357 | 0.171083256461542 | 0.672327672327672 | 4.62487058933275 | 4.79595384579429 | 0.76969696969697 | 0.915151515151515 | 0.84048770167482 | SC;SQ;UA | SC;SQ;UA | NA |
| 50 | 1284 | 101284 | 0.19269376066597 | 0.554445554445554 | 0.29107128543085 | 0.554445554445554 | 4.62487058933275 | 4.3337993039019 | 0.709090909090909 | 0.709090909090909 | 0.858865925136353 | UA;ST;SW | UA;SE;SW | NA |
| 51 | 1285 | 101285 | 0.179430755712468 | 0.608391608391608 | 0.0136226948125344 | 0.974025974025974 | 4.62487058933275 | 4.61124789452022 | 0.951515151515151 | 0.890909090909091 | 0.889851646188759 | UA;SC;SQ | UA;SC;SQ | NA |
| 52 | 1286 | 101286 | 0.18214850542235 | 0.607392607392607 | 0.608917295319893 | 0.316683316683317 | 4.62487058933275 | 5.23378788465264 | 0.878787878787879 | 0.939393939393939 | 0.854560941401842 | UA;SQ;ST | UA;SQ;SC | NA |
| 53 | 1287 | 101287 | 0.326269896301895 | 0.018981018981019 | 0.000792683038332243 | 1 | 4.62487058933275 | 4.62566327237108 | 0.951515151515151 | 0.915151515151515 | 0.732633049901443 | UA;SC;SE | UA;SQ;SC | NA |
| 54 | 1288 | 101288 | 0.216067771596257 | 0.35964035964036 | 0.012409774200739 | 0.972027972027972 | 4.62487058933275 | 4.61246081513201 | 0.951515151515151 | 0.963636363636364 | 0.867193645656049 | UA;SC;SQ | UA;SC;SQ | NA |
| 55 | 1289 | 101289 | 0.295299518374024 | 0.040959040959041 | 0.27141832428381 | 0.58041958041958 | 4.62487058933275 | 4.35345226504894 | 0.963636363636364 | 0.963636363636364 | 0.85063796133238 | UA;SC;SQ | UA;SC;SQ | NA |
| 56 | 1290 | 101290 | 0.207113743666738 | 0.390609390609391 | 0.149883798285678 | 0.656343656343656 | 4.62487058933275 | 4.77475438761843 | 0.76969696969697 | 0.963636363636364 | 0.83937862289741 | SQ;SC;ST | SQ;SC;UA | NA |
| 57 | 1291 | 101291 | 0.296993413336201 | 0.046953046953047 | 1.28762336830522 | 0.00899100899100899 | 4.62487058933275 | 5.91249395763797 | 0.890909090909091 | 0.939393939393939 | 0.760570770829207 | SC;UA;ST | UA;SQ;SC | NA |
| 58 | 1292 | 101292 | 0.198879909489978 | 0.451548451548452 | 0.125156160364225 | 0.796203796203796 | 4.62487058933275 | 4.75002674969698 | 0.890909090909091 | 0.915151515151515 | 0.901791784106991 | SE;UA;SC | SC;UA;SQ | NA |
| 59 | 1293 | 101293 | 0.255602391549766 | 0.132867132867133 | 0.842055851460167 | 0.0839160839160839 | 4.62487058933275 | 5.46692644079292 | 0.866666666666667 | 0.878787878787879 | 0.792677589215596 | SC;ST;UA | UA;SC;SE | NA |
| 60 | 1294 | 101294 | 0.187125717502729 | 0.521478521478521 | 0.0922137412932136 | 0.771228771228771 | 4.62487058933275 | 4.71708433062597 | 0.878787878787879 | 0.793939393939394 | 0.853788345264159 | UA;SE;ST | UA;SE;SW | NA |
| 61 | 1295 | 101295 | 0.298362638097013 | 0.034965034965035 | 0.0759863191149988 | 0.815184815184815 | 4.62487058933275 | 4.54888427021775 | 0.915151515151515 | 0.915151515151515 | 0.844840137134548 | SC;UA;SQ | SC;SQ;UA | NA |
| 62 | 1296 | 101296 | 0.285896691525336 | 0.0549450549450549 | 0.255382173369676 | 0.480519480519481 | 4.62487058933275 | 4.36948841596308 | 0.963636363636364 | 0.878787878787879 | 0.693297690723082 | SC;UA;SE | SC;UA;SE | NA |
| 63 | 1297 | 101297 | 0.345228698445318 | 0.00699300699300699 | 1.84429371121112 | 0.00699300699300699 | 4.62487058933275 | 6.46916430054387 | 0.660606060606061 | 0.83030303030303 | 0.643267359938373 | ST;SC;SE | SC;UA;SE | NA |
| 64 | 1298 | 101298 | 0.204102283788842 | 0.384615384615385 | 0.125213398710204 | 0.735264735264735 | 4.62487058933275 | 4.75008398804296 | 0.866666666666667 | 0.975757575757576 | 0.843026097564883 | UA;SC;SQ | UA;SQ;SC | NA |
| 65 | 1299 | 101299 | 0.237513729481498 | 0.178821178821179 | 0.824566576437661 | 0.0699300699300699 | 4.62487058933275 | 5.44943716577041 | 0.442424242424242 | 0.915151515151515 | 0.814879954073141 | ST;SC;PD | UA;SQ;SC | NA |
| 66 | 1300 | 101300 | 0.16474707034211 | 0.698301698301698 | 0.217808577826597 | 0.627372627372627 | 4.62487058933275 | 4.40706201150615 | 0.915151515151515 | 1 | 0.819390480552991 | SC;UA;SQ | UA;SQ;SC | NA |
| 67 | 1301 | 101301 | 0.239399738705273 | 0.176823176823177 | 0.371609779104594 | 0.352647352647353 | 4.62487058933275 | 4.99648036843735 | 0.733333333333333 | 0.915151515151515 | 0.784813925715346 | SC;SQ;MA | SC;SQ;UA | NA |
| 68 | 1302 | 101302 | 0.159131818565952 | 0.756243756243756 | 0.343204302693057 | 0.444555444555445 | 4.62487058933275 | 4.96807489202581 | 0.648484848484848 | 0.890909090909091 | 0.846909200895522 | UA;SW;ST | UA;SE;SC | NA |
| 69 | 1303 | 101303 | 0.350275531939465 | 0.012987012987013 | 1.07772875235552 | 0.114885114885115 | 4.62487058933275 | 5.70259934168827 | 0.757575757575757 | 0.878787878787879 | 0.782999865717739 | SQ;ST;UA | UA;SC;SQ | NA |
| 70 | 1304 | 101304 | 0.179912970504971 | 0.643356643356643 | 0.243063800186101 | 0.561438561438561 | 4.62487058933275 | 4.86793438951885 | 0.878787878787879 | 0.854545454545454 | 0.790232251020378 | UA;SC;ST | SC;UA;SQ | NA |
| 71 | 1305 | 101305 | 0.239916560988573 | 0.192807192807193 | 0.606251895740898 | 0.111888111888112 | 4.62487058933275 | 5.23112248507365 | 0.866666666666667 | 0.878787878787879 | 0.831129223680038 | SC;SQ;UA | SC;SQ;UA | NA |
| 72 | 1306 | 101306 | 0.283432026100912 | 0.041958041958042 | 0.0892312210741588 | 0.86013986013986 | 4.62487058933275 | 4.53563936825859 | 0.878787878787879 | 0.951515151515151 | 0.857292776584882 | SC;UA;SQ | UA;SC;SQ | NA |
| 73 | 1307 | 101307 | 0.186011464621543 | 0.572427572427572 | 0.898753744047091 | 0.0759240759240759 | 4.62487058933275 | 5.52362433337984 | 0.806060606060606 | 0.987878787878788 | 0.86874539632731 | UA;SW;SQ | UA;SQ;SC | NA |
| 74 | 1308 | 101308 | 0.279179429200821 | 0.0609390609390609 | 1.05320185566612 | 0.02997002997003 | 4.62487058933275 | 5.67807244499887 | 0.672727272727273 | 0.854545454545454 | 0.842437988276202 | SW;SC;UA | SC;UA;SQ | NA |
| 75 | 1309 | 101309 | 0.224943428495095 | 0.237762237762238 | 0.0316798734301518 | 0.935064935064935 | 4.62487058933275 | 4.5931907159026 | 0.903030303030303 | 0.903030303030303 | 0.859778745961731 | SC;UA;ST | SC;UA;SQ | NA |
| 76 | 1310 | 101310 | 0.286278478052223 | 0.0539460539460539 | 1.04238012972556 | 0.025974025974026 | 4.62487058933275 | 5.66725071905831 | 0.781818181818182 | 0.915151515151515 | 0.823130298554768 | UA;SC;SE | UA;SE;SC | NA |
| 77 | 1311 | 101311 | 0.229696357970898 | 0.240759240759241 | 0.515051285807037 | 0.298701298701299 | 4.62487058933275 | 5.13992187513979 | 0.975757575757576 | 0.975757575757576 | 0.858611459889341 | UA;SE;SC | UA;SQ;SC | NA |
| 78 | 1312 | 101312 | 0.13178259774948 | 0.918081918081918 | 0.172857267605098 | 0.663336663336663 | 4.62487058933275 | 4.45201332172765 | 0.975757575757576 | 0.927272727272727 | 0.853696697486822 | SC;UA;SE | SC;UA;SE | NA |
| 79 | 1313 | 101313 | 0.289206165800923 | 0.0629370629370629 | 1.91982410057049 | 0.00599400599400599 | 4.62487058933275 | 6.54469468990324 | 0.709090909090909 | 0.806060606060606 | 0.782430524214814 | UA;SC;SQ | UA;SQ;SE | NA |
| 80 | 1314 | 101314 | 0.344260425206449 | 0.003996003996004 | 1.62909501070421 | 0.00799200799200799 | 4.62487058933275 | 6.25396560003697 | 0.757575757575757 | 0.939393939393939 | 0.794733996729159 | ST;SC;SE | UA;SQ;SE | NA |
| 81 | 1315 | 101315 | 0.352145586729175 | 0.00699300699300699 | 2.19360253713132 | 0.000999000999000999 | 4.62487058933275 | 6.81847312646407 | 0.466666666666667 | 0.806060606060606 | 0.68832923304378 | UA;MA;SC | UA;SC;SQ | NA |
| 82 | 1316 | 101316 | 0.227036402717711 | 0.302697302697303 | 0.376108188410891 | 0.424575424575425 | 4.62487058933275 | 5.00097877774364 | 0.660606060606061 | 0.903030303030303 | 0.816685025139079 | UA;ST;SE | UA;SE;SC | NA |
| 83 | 1317 | 101317 | 0.368751394786879 | 0.002997002997003 | 2.24741090642109 | 0.000999000999000999 | 4.62487058933275 | 6.87228149575384 | 0.793939393939394 | 0.854545454545454 | 0.521339853357414 | ST;SC;UA | UA;ST;SC | NA |
| 84 | 1318 | 101318 | 0.24201141748269 | 0.160839160839161 | 0.231782599377075 | 0.588411588411588 | 4.62487058933275 | 4.39308798995568 | 0.83030303030303 | 0.915151515151515 | 0.786443897473055 | SQ;SC;UA | SQ;SC;UA | NA |
| 85 | 1319 | 101319 | 0.204716828721944 | 0.408591408591409 | 0.238703862724488 | 0.586413586413586 | 4.62487058933275 | 4.38616672660826 | 0.806060606060606 | 0.890909090909091 | 0.864757759048617 | UA;SQ;ST | UA;SQ;ST | NA |
| 86 | 1320 | 101320 | 0.309298365826585 | 0.024975024975025 | 1.72904258278948 | 0.004995004995005 | 4.62487058933275 | 6.35391317212224 | 0.866666666666667 | 0.878787878787879 | 0.733961297589622 | UA;ST;SQ | UA;SQ;SE | NA |
| 87 | 1321 | 101321 | 0.294387916071366 | 0.050949050949051 | 0.554376429979913 | 0.228771228771229 | 4.62487058933275 | 5.17924701931267 | 0.878787878787879 | 0.866666666666667 | 0.762253256344837 | SE;SC;UA | SC;SE;UA | NA |
| 88 | 1322 | 101322 | 0.245950105665618 | 0.171828171828172 | 0.114173988279344 | 0.781218781218781 | 4.62487058933275 | 4.51069660105341 | 0.927272727272727 | 0.951515151515151 | 0.758926789921955 | UA;SQ;SC | UA;SQ;SC | NA |
| 89 | 1323 | 101323 | 0.25981342586802 | 0.104895104895105 | 0.331859169660506 | 0.392607392607393 | 4.62487058933275 | 4.95672975899326 | 0.745454545454545 | 0.963636363636364 | 0.855693667147271 | SC;SQ;SE | SQ;SC;UA | NA |
| 90 | 1324 | 101324 | 0.358551099850568 | 0.003996003996004 | 1.40444316137971 | 0.028971028971029 | 4.62487058933275 | 6.02931375071247 | 0.587878787878788 | 0.939393939393939 | 0.789756402102792 | PD;UA;SQ | SC;UA;SQ | NA |
| 91 | 1325 | 101325 | 0.247742705786852 | 0.154845154845155 | 1.78066523830277 | 0.00699300699300699 | 4.62487058933275 | 6.40553582763552 | 0.757575757575757 | 0.83030303030303 | 0.728478442516153 | SE;SC;MA | SE;SC;UA | NA |
| 92 | 1326 | 101326 | 0.248865931519524 | 0.184815184815185 | 1.1208747376377 | 0.03996003996004 | 4.62487058933275 | 5.74574532697045 | 0.563636363636364 | 0.915151515151515 | 0.827527789222132 | SE;UA;SW | SQ;SC;UA | NA |
| 93 | 1327 | 101327 | 0.207113743666738 | 0.365634365634366 | 0.00905110772983164 | 0.978021978021978 | 4.62487058933275 | 4.61581948160292 | 0.975757575757576 | 0.915151515151515 | 0.79034090713627 | UA;SC;SQ | SC;UA;SQ | NA |
| 94 | 1328 | 101328 | 0.207113743666738 | 0.387612387612388 | 0.274319890540881 | 0.572427572427572 | 4.62487058933275 | 4.89919047987363 | 0.939393939393939 | 0.915151515151515 | 0.883332519439155 | SC;SE;UA | SC;UA;SQ | NA |
| 95 | 1329 | 101329 | 0.229163192927344 | 0.231768231768232 | 0.0780679438839815 | 0.805194805194805 | 4.62487058933275 | 4.70293853321673 | 0.939393939393939 | 0.951515151515151 | 0.903190619804829 | UA;SE;ST | UA;SE;SQ | NA |
| 96 | 1330 | 101330 | 0.207113743666738 | 0.385614385614386 | 0.13990682904814 | 0.745254745254745 | 4.62487058933275 | 4.48496376028461 | 0.975757575757576 | 0.915151515151515 | 0.784263714143971 | SC;UA;SE | SC;UA;SE | NA |
| 97 | 1331 | 101331 | 0.330947196364127 | 0.00799200799200799 | 0.351649003982625 | 0.416583416583417 | 4.62487058933275 | 4.97651959331538 | 0.721212121212121 | 0.890909090909091 | 0.765771294411831 | SC;SE;SW | SC;UA;SQ | NA |
| 98 | 1332 | 101332 | 0.300080565575815 | 0.036963036963037 | 0.112258702816807 | 0.732267732267732 | 4.62487058933275 | 4.51261188651594 | 0.854545454545454 | 0.903030303030303 | 0.780460301369267 | UA;ST;SQ | UA;SQ;SC | NA |
| 99 | 1333 | 101333 | 0.239991762410844 | 0.147852147852148 | 0.023663713239209 | 0.957042957042957 | 4.62487058933275 | 4.60120687609354 | 0.939393939393939 | 0.890909090909091 | 0.760665738227098 | UA;SE;ST | UA;SE;SC | NA |
| 100 | 1334 | 101334 | 0.337389767356176 | 0.011988011988012 | 0.106991710871425 | 0.813186813186813 | 4.62487058933275 | 4.73186230020418 | 0.963636363636364 | 0.927272727272727 | 0.834258795962269 | SC;UA;SQ | SC;UA;SQ | NA |
